# Dr. Ambrose Lawrence

**Published:** 1893-06

**Authors:** 


### DR. AMBROSE LAWRENCE.

Dr. Ambrose Lawrence, of Boston, died April 23, 1893. Dr. Lawrence was the originator of the well-known Lawrence's amalgam. Its introduction in 1851, at a time when amalgams were regarded with no favor, had a tendency to place him in a peculiar position in relation to the active workers of his day; but the dental profession in time changed its opinion entirely in regard to this material, and probably in a great measure lost some of the feeling originally felt towards those who manufactured and persistently advocated its use.

Dr. Lawrence had his personal peculiarities, but was pleasant socially and professionally, and in former years active in dental associations. He for a long period apparently kept apart from active organizations, and has only been known to the present generation of dentists as the manufacturer of the material bearing his name.

He had reached the age of seventy-seven years at the time of his death, and had continued almost to the period of his sudden transition in active practice.

